# Quality of life analysis of patients treated with cetuximab or cisplatin for locoregionally advanced squamous cell carcinoma of head and neck in the United States

**DOI:** 10.1186/s12955-020-01424-x

**Published:** 2020-06-22

**Authors:** Himani Aggarwal, Rajeshwari S. Punekar, Li Li, Gebra Cuyun Carter, Mark S. Walker

**Affiliations:** 1 Global Patient Outcomes and Real World Evidence, Eli Lilly and Company, Indianapolis, IN USA; 2 Vector Oncology, Memphis, TN USA; 3 Statistics, R&G PharmaStudies Co., Ltd., Somerset, NJ USA

**Keywords:** Quality of life, Advanced squamous cell carcinoma of the head and neck, Cetuximab, Cisplatin, Radiation therapy

## Abstract

**Background:**

To compare quality of life of patients treated with cetuximab with or without radiation therapy (±RT) vs. cisplatin±RT for locoregionally advanced squamous cell carcinoma of the head and neck (SCCHN) in the real-world setting.

**Methods:**

In this retrospective observational study, electronic medical records and Patient Care Monitor (PCM) survey data from the Vector Oncology Data Warehouse were utilized from adult patients in the United States who received initial treatment with cetuximab±RT or cisplatin±RT for locoregionally advanced SCCHN between January 1, 2007 and January 1, 2017. Quality of life was assessed using PCM index scores and individual PCM items. Cetuximab±RT and cisplatin±RT cohorts were balanced using propensity score weighting. Linear mixed models were used to assess the impact of baseline demographic and clinical characteristics on PCM endpoints.

**Results:**

Of 531 patients with locoregionally advanced SCCHN, 187 received cetuximab±RT, and 344 received cisplatin±RT. Before propensity score weighting, the cetuximab±RT cohort was older (mean [SD] age of 63.9 [9.6] years vs. 57.4 [8.6] years), and more likely to be white (82.4% vs. 72.4%) compared to the cisplatin±RT cohort. After propensity score weighting, the two cohort subsamples (cetuximab±RT, *N* = 60; cisplatin±RT, *N* = 177) with PCM data showed no significant differences in General Physical Symptoms, Treatment Side Effects, Impaired Ambulation, or Impaired Performance index scores. Patients in the cetuximab±RT cohort had higher Acute Distress index (*p* = 0.023), Despair index (*p* = 0.011), and rash (*p* = 0.003) scores but lower numbness/tingling scores (*p* = 0.022) than patients in the cisplatin±RT cohort.

**Conclusions:**

Significant group differences were observed in this comparative analysis, as the cetuximab±RT cohort had significantly higher Acute Distress index, Despair index, and rash scores compared with the cisplatin±RT cohort but lower numbness/tingling scores. These patterns of symptoms appear consistent with previously reported symptoms associated with the treatment of SCCHN.

## Background

Head and neck cancer includes a wide range of malignant tumors originating in the upper aerodigestive tract, including the oral cavity, pharynx, larynx, paranasal sinuses and nasal cavity, and salivary glands [1]. It is the sixth most common cancer worldwide, representing 2.5% of new cancer cases and 1.9% of cancer deaths annually [2]. Approximately 4% of cancer diagnoses in the United States (US) are head and neck cancers, with 64,690 new cases and 13,740 deaths in 2017 [3]. More than 90% of head and neck cancers are squamous cell carcinomas that arise in the squamous cells lining the mucosal surfaces inside the head and neck [4]. The most common causes of head and neck cancers are tobacco and alcohol use [5]. Human papillomavirus (HPV) has been shown to cause oropharyngeal cancer [6]. HPV-negative cancers are more strongly associated with tobacco and alcohol use, whereas HPV-positive cancers are typically related to sexual behavior [6]. Relative to HPV-negative malignancies, HPV-positive cancers have more favorable prognoses [6–8]. The incidence of HPV-positive oropharyngeal squamous cell carcinoma is rising, whereas the incidence of HPV-negative head and neck cancers is declining [6, 9].

Sixty six percent of people with head and neck cancers are diagnosed at advanced stages (III or IV) [10]. The preferred treatment approach for patients with locally advanced disease remains concurrent cisplatin and radiotherapy [11]; however, a disadvantage of high-dose cisplatin is increased acute and late toxic effects [12]. Cetuximab may offer a less toxic option for patients unfit for high-dose cisplatin [13].

The effects of squamous cell carcinoma of the head and neck (SCCHN) and its treatment on patients’ lives can be deleterious. Patients who undergo chemoradiation therapy (CRT) suffer from significant physical symptoms such as xerostomia, mucositis, dysphagia, difficulty swallowing, and psychological symptoms such as social isolation and depression which adversely affect their quality of life (QoL) [14–16] making QoL an important consideration for treatment selection.

Reports of QoL among real-world patients with advanced SCCHN remain limited. This study addresses these gaps through in-depth analysis of the QoL of patients with advanced stage SCCHN treated in the community oncology setting. The objective of this study was to compare the QoL of patients treated with cetuximab with or without radiation therapy (±RT) vs. cisplatin±RT for locoregionally advanced SCCHN in a real-world setting.

## Methods

### Study design

This retrospective observational study utilized data from the Vector Oncology Data Warehouse, which contains electronic medical records and Patient Care Monitor (PCM) survey data collected as part of routine clinical care at participating practices in the US. The study sample included adult patients who received initial treatment with cetuximab±RT or cisplatin±RT for locoregionally advanced SCCHN.

### Study outcomes and variables

The main outcomes of interest were QoL and select patient-reported symptoms of advanced stage SCCHN patients treated with cetuximab±RT or cisplatin±RT. These outcomes were analyzed using PCM (version 2.0) survey data. The PCM is a validated survey instrument that assesses oncology-related symptoms, using an 11-point Likert scale. Individual items are rated from 0 (not a problem) to 10 (as bad as possible), indicating the degree to which the symptom has been a problem in the past week [17–19]. The instrument assesses 86 individual symptoms and generates 6 index scores that describe global function [19]. The PCM has been shown to be valid [17, 18] and used in numerous studies of QoL and symptom burden in cancer patient populations [20–23].

The 6 PCM index scores included General Physical Symptoms (physical symptoms associated with cancer, but not necessarily with systemic therapy), Treatment Side Effects (symptoms more commonly associated with systemic therapy), Acute Distress (a measure of anxiety), Despair (a measure of depressive symptoms), Impaired Ambulation (functional impairment tied to mobility), and Impaired Performance (other functional impairment). PCM index scores are standardized to T-scores, with a higher index score indicating worse symptoms. Select PCM items representing symptoms commonly experienced by patients with SCCHN treated with CRT included trouble swallowing, dry mouth, numbness/tingling, rash, weight loss, and mouth sores/ulcers. PCM surveys were offered at every patient visit, assuming visits were greater than 1 week apart.

Demographic and clinical variables included in the analysis were age, gender, race, ethnicity, body mass index (BMI), type of insurance, region of residence, stage of disease at initial diagnosis, tumor location at initial diagnosis, HPV status, tumor suppressor protein p16 status, placement of feeding tube, smoking status, alcohol usage, Eastern Cooperative Oncology Group (ECOG) performance status, composite performance status, comorbidities, and weighted index of comorbid conditions.

### Patients

Eligible patients were those diagnosed with locoregionally advanced (stage III/IVA/IVB) SCCHN (ICD-9-CM codes: 140–149 or 161, or ICD-10-CM codes: C00-C14 or C32) between January 1, 2007 and January 1, 2017 who received cetuximab±RT or cisplatin±RT as initial treatment. Patients who met the inclusion criteria were stratified into the cetuximab±RT or cisplatin±RT cohort. Excluded patients were those who received both cetuximab and cisplatin as initial treatment in the locoregionally advanced setting or those who had a record of treatment for another cancer during the 6 months prior to diagnosis of advanced SCCHN (with the exception of nonmelanoma skin cancer and carcinoma in situ).

### Statistical methods

Patients’ demographic and clinical characteristics were compared between cetuximab±RT and cisplatin±RT cohorts using t-test (or Wilcoxon Rank-Sum test, if normality did not hold) for continuous variables or Chi-square (or Fisher’s Exact test, if more than 20% of expected frequencies were less than 5) for categorical variables.

For comparative analysis, cetuximab±RT and cisplatin±RT cohorts were balanced using propensity score methods. To generate propensity scores, a propensity score model (PSM) was developed using a main effects logistic regression model, in which treatment (cetuximab±RT or cisplatin±RT) was the dependent variable and covariates included age, race, BMI, type of insurance, patient’s region of residence, tumor site, HPV and p16 test results, feeding tube placement status, smoking status, alcohol consumption status, impairment status, and weighted index of comorbid conditions. The suitability of the PSM was assessed by reviewing the distribution of propensity scores, and trimming the sample as needed to exclude cases for which class membership was highly determined (propensity score < 5% or > 80%). Propensity scores generated through the PSM were used to generate inverse probability of treatment weights (IPTW). Because case weighting altered the implied study sample, weights were rescaled so that the sum of the weights in the trimmed sample was equal to the unweighted trimmed sample size. The balance between the 2 cohorts was verified by statistically comparing the baseline patient characteristics across the weighted samples. Nonsignificance (*p* > 0.05) of group differences on weighted comparisons was interpreted as evidence of group balance, given the propensity score. The probability of a type I error, α, was set at 0.05. SAS 9.2 (SAS Institute, Cary, NC, US) was used for analyses.

### Linear mixed model

Linear mixed models (LMM) were used to examine differences in PCM endpoints between cetuximab±RT and cisplatin±RT cohorts after applying IPTW. Models assessed main effects of treatment and time since diagnosis and interaction effect of treatment with time since diagnosis. Baseline demographic and clinical characteristics were controlled in models. Methods generally followed those described by Cnaan et al. [24] and Littell et al. [25]. To characterize the clinical significance of the LMM effect estimates, this study used a 1-point difference for individual PCM items and a 3-point difference for PCM index scores, based on findings that 5–10% of instrument range is the threshold for clinical meaningfulness [26]. No correction for multiple testing was done across models.

## Results

### Study sample

The study accrued 706 patients in total and addressed research questions that go beyond the scope of this manuscript [27]. The focus of the analysis reported here is patients who received cetuximab or cisplatin in the locoregionally advanced setting and who were retained after sample trimming that occurred as part of PSM (*N* = 531). Demographic and clinical characteristics of this sample are presented below. Of the 531 patients, 237 provided PCM data and contributed to analysis of the PCM endpoints.

### Demographic and clinical characteristics

#### Before propensity score weighting

Prior to propensity score weighting, the trimmed sample of 531 patients included 187 patients in the cetuximab±RT cohort and 344 patients in the cisplatin±RT cohort, with differences between the cohorts with regard to age, region of residence, p16 testing status, alcohol use, and weighted index of comorbid conditions [28] (Table 1). The cetuximab±RT cohort was generally older than the cisplatin±RT cohort (mean age: 63.9 vs. 57.4 years; *p*<0.0001). Race varied across the cohorts (*p* = 0.075), an effect that appeared to be driven by a higher rate of white patients in the cetuximab±RT cohort (82.4% vs. 72.4%). The type of insurance used by patients across cetuximab±RT and cisplatin±RT cohorts varied (*p* = 0.054), a difference that is likely an artifact of the age difference between cohorts. Region of residence varied significantly across the two cohorts (*p* = 0.005), an effect potentially driven by a higher rate of residency in southern US in the cisplatin±RT cohort (88.4% vs. 78.6%). The cisplatin±RT cohort had a significantly greater percentage of patients who were tested for p16 status compared to the cetuximab±RT cohort (38.1% vs. 23.0%; *p* = 0.0004). Numerically greater proportions of patients in the cisplatin±RT than in the cetuximab±RT cohort were current smokers (45.1% vs. 38.5%) and alcohol users and abusers (50.9% vs. 42.8%), whereas numerically greater proportions of patients in the cetuximab±RT than in the cisplatin±RT cohort were past smokers (43.3% vs. 35.8%) and past alcohol users and abusers (32.1% vs. 20.6%). The weighted index of comorbid conditions varied significantly  between the two cohorts (*p*<0.0001), an effect possibly driven by a higher rate of patients with no comorbid conditions in the cisplatin±RT cohort (64.0% vs. 44.9%).

**Table 1:** Demographic and clinical characteristics

	Before propensity score weighting			After propensity score weighting with rescaled IPTW^*^		
Characteristic	Cetuximab±RT (*N* = 187)	Cisplatin±RT (*N* = 344)	*P*-value^a^	Cetuximab±RT (*N* = 263)	Cisplatin±RT (*N* = 268)	*P*-value^a^
Age, years			<0.0001			0.902
Mean (SD)	63.9 (9.62)	57.4 (8.58)		59.3 (11.69)	59.4 (7.70)	
Male, n (%)	160 (85.6)	281 (81.7)	0.256	222 (84.4)	219 (82.0)	0.453
Race, n (%)			0.075			0.298
White	154 (82.4)	249 (72.4)		208 (78.9)	204 (76.1)	
Black or African American	30 (16.0)	83 (24.1)		52 (19.9)	54 (20.1)	
Other	1 (0.5)	5 (1.5)		1 (0.4)	4 (1.5)	
Undocumented	2 (1.1)	7 (2.0)		2 (0.8)	6 (2.3)	
Ethnicity, n (%)			0.543			0.175
Hispanic or Latino	0 (0)	2 (0.6)		0 (0.0)	2 (0.7)	
Not Hispanic or Latino	187 (100.0)	342 (99.4)		263 (100.0)	266 (99.3)	
BMI, kg/m^2^			0.857			0.773
Mean (SD)	26.7 (6.50)	26.8 (6.27)		27.0 (7.45)	26.8 (5.41)	
Median	25.7	26.0		26.6	26.0	
Min., max.	15.55, 59.41	13.62, 49.58		15.55, 59.41	13.62, 49.58	
Type of insurance, n (%)			0.054			0.877
Private, no public	48 (25.7)	127 (36.9)		84 (32.1)	91 (33.8)	
Public, no private	63 (33.7)	102 (29.7)		89 (33.9)	83 (30.9)	
Public and private	66 (35.3)	98 (28.5)		77 (29.1)	80 (29.7)	
Other	1 (0.5)	6 (1.7)		3 (1.0)	5 (1.9)	
Unknown or undocumented	9 (4.8)	11 (3.2)		10 (3.9)	10 (3.6)	
US region of residence, n (%)			0.005			0.627
Northeast	1 (0.5)	2 (0.6)		1 (0.5)	1 (0.5)	
South	147 (78.6)	304 (88.4)		227 (86.3)	226 (84.3)	
Midwest	39 (20.9)	37 (10.8)		35 (13.2)	39 (14.6)	
West	0 (0.0)	1 (0.3)		0 (0.0)	2 (0.6)	
Stage of disease at initial diagnosis, n (%)			0.228			0.388
I	3 (1.6)	2 (0.6)		5 (2.0)	1 (0.5)	
II	3 (1.6)	4 (1.2)		2 (0.9)	5 (1.7)	
III	55 (29.4)	92 (26.7)		74 (28.1)	71 (26.6)	
IV	10 (5.3)	9 (2.6)		13 (5.0)	7 (2.5)	
IVA	98 (52.4)	202 (58.7)		140 (53.0)	154 (57.7)	
IVB	9 (4.8)	26 (7.6)		18 (7.0)	22 (8.4)	
Other	1 (0.5)	2 (0.6)		1 (0.3)	1 (0.5)	
Undocumented	8 (4.3)	7 (2.0)		10 (3.8)	6 (2.1)	
Tumor location at initial diagnosis, n (%)						
Oral cavity	26 (13.9)	38 (11.0)	0.334	34 (12.8)	32 (12.0)	0.780
Pharynx	119 (63.6)	220 (64.0)	0.942	170 (64.6)	171 (63.9)	0.868
Paranasal sinuses	0 (0.0)	2 (0.6)	0.543	0 (0.0)	3 (1.0)	0.110
Salivary glands	1 (0.5)	4 (1.2)	0.661	1 (0.3)	3 (1.1)	0.250
Larynx	40 (21.4)	81 (23.5)	0.572	58 (22.0)	60 (22.4)	0.899
Unknown or undocumented	3 (1.6)	3 (0.9)	0.430	4 (1.4)	2 (0.8)	0.494
HPV results, n (%)			0.361			0.084
Positive	21 (70.0)	24 (60.0)		37 (82.9)	16 (61.5)	
Negative	7 (23.3)	15 (37.5)		6 (13.1)	9 (35.8)	
Undocumented	2 (6.7)	1 (2.5)		2 (3.9)	1 (2.7)	
Tested for p16 status, n (%)			0.0004			0.001
No/undocumented	144 (77.0)	213 (61.9)		200 (76.1)	169 (63.2)	
Yes	43 (23.0)	131 (38.1)		63 (23.9)	98 (36.8)	
P16 results, n (%)			0.817			0.104
Positive	30 (69.8)	84 (64.1)		53 (77.7)	66 (62.4)	
Negative	12 (27.9)	44 (33.6)		14 (20.7)	38 (35.6)	
Undocumented	1 (2.3)	3 (2.3)		1 (1.6)	2 (2.1)	
Placement of feeding tube, n (%)			0.398			0.967
Not placed	66 (35.3)	109 (31.7)		86 (32.5)	88 (32.7)	
Placed	121 (64.7)	235 (68.3)		178 (67.5)	180 (67.3)	
Smoking status, n (%)			0.073			0.318
Current	72 (38.5)	155 (45.1)		111 (42.3)	120 (44.8)	
Past	81 (43.3)	123 (35.8)		104 (39.6)	97 (36.4)	
Never	32 (17.1)	66 (19.2)		45 (17.1)	50 (18.8)	
Unknown or undocumented	2 (1.1)	0 (0.0)		3 (1.0)	0 (0.0)	
Alcohol use, n (%)			0.026			0.097
Current abuse	13 (7.0)	35 (10.2)		19 (7.2)	24 (9.1)	
Current use	67 (35.8)	140 (40.7)		99 (37.7)	108 (40.5)	
No current use, no past use	46 (24.6)	87 (25.3)		67 (25.5)	66 (24.8)	
Past use, no current use	31 (16.6)	33 (9.6)		45 (17.0)	29 (11.0)	
Past abuse	29 (15.5)	38 (11.0)		32 (12.1)	31 (11.4)	
Unknown or undocumented	1 (0.5)	11 (3.2)		1 (0.5)	8 (3.2)	
ECOG PS, n (%)			0.355			0.537
0	45 (24.1)	88 (25.6)		74 (28.1)	65 (24.4)	
1	34 (18.2)	47 (13.7)		40 (15.3)	37 (13.8)	
2	7 (3.7)	13 (3.8)		6 (2.2)	12 (4.4)	
3	2 (1.1)	1 (0.3)		2 (0.7)	1 (0.3)	
4	1 (0.5)	0 (0.0)		1 (0.3)	0 (0.0)	
Undocumented	98 (52.4)	195 (56.7)		141 (53.5)	153 (57.0)	
Composite performance status, n (%)			0.346			0.663
Impaired	11 (5.9)	14 (4.1)		10 (4.0)	13 (4.7)	
Not impaired	176 (94.1)	330 (95.9)		253 (96.0)	255 (95.3)	
Weighted index of comorbid conditions, n (%)			<0.0001			0.689
0	84 (44.9)	220 (64.0)		161 (61.3)	155 (57.8)	
1	62 (33.2)	93 (27.0)		67 (25.4)	80 (30.0)	
2	23 (12.3)	22 (6.4)		21 (8.1)	21 (7.9)	
3–5	17 (9.1)	9 (2.6)		13 (4.9)	12 (4.4)	
6+	1 (0.5)	0 (0.0)		1 (0.3)	0 (0.0)	

*Propensity score modeling led to exclusion of 33 cases within the cetuximab±RT group (trimmed *N* = 187) and 12 cases within the cisplatin±RT group (trimmed *N* = 344), leaving an unweighted total sample of 531 patients. Weighting of cases with IPTW altered the implied total sample; therefore, cases were rescaled to produce a sum of weights equal to the trimmed sample of 531. This resulted in weighted study samples across groups that were nearly equal (263 cetuximab±RT, 268 cisplatin±RT)

*BMI* body mass index, *ECOG PS* Eastern Cooperative Oncology Group performance status, *HPV* human papillomavirus, *IPTW* inverse probability of treatment weight, *Max* maximum, *min* minimum, *PSM* propensity score model, *RT* radiation therapy, *SD* standard deviation, *US* United States

^a^*P*-values are from t-test (or Wilcoxon Rank-Sum test, if normality does not hold) for continuous variables or Chi-squared (or Fisher’s Exact test, if expected frequencies are less than 5) for categorical variables

#### After propensity score weighting

Propensity score modeling was used to account for potential treatment selection effects in the comparative analysis. Weighting of cases with IPTW altered the implied total sample; therefore, case weights were rescaled to produce a sum of weights equal to the trimmed sample of 531. This resulted in weighted study samples across groups that were nearly equal (263 cetuximab±RT, 268 cisplatin±RT), and in which baseline differences were no longer significant, except p16 testing status. Table 1 shows baseline characteristics in the trimmed sample, by group, before and after propensity score weighting.

### Quality of life

Rescaled weighting of cases with IPTW, as described above, was employed in all comparisons of PCM endpoints using the subset of patients in each cohort who provided PCM survey data. This included rescaled samples of 60 patients in the cetuximab±RT cohort and 177 patients in the cisplatin±RT cohort.

Comparison of patients with and without PCM surveys (data not shown) revealed no meaningful difference between patients with and without survey data, except minor demographic differences associated with the geographic region (southern US) where the surveys were more widely administered.

#### General physical symptoms

**Fig. 1:**
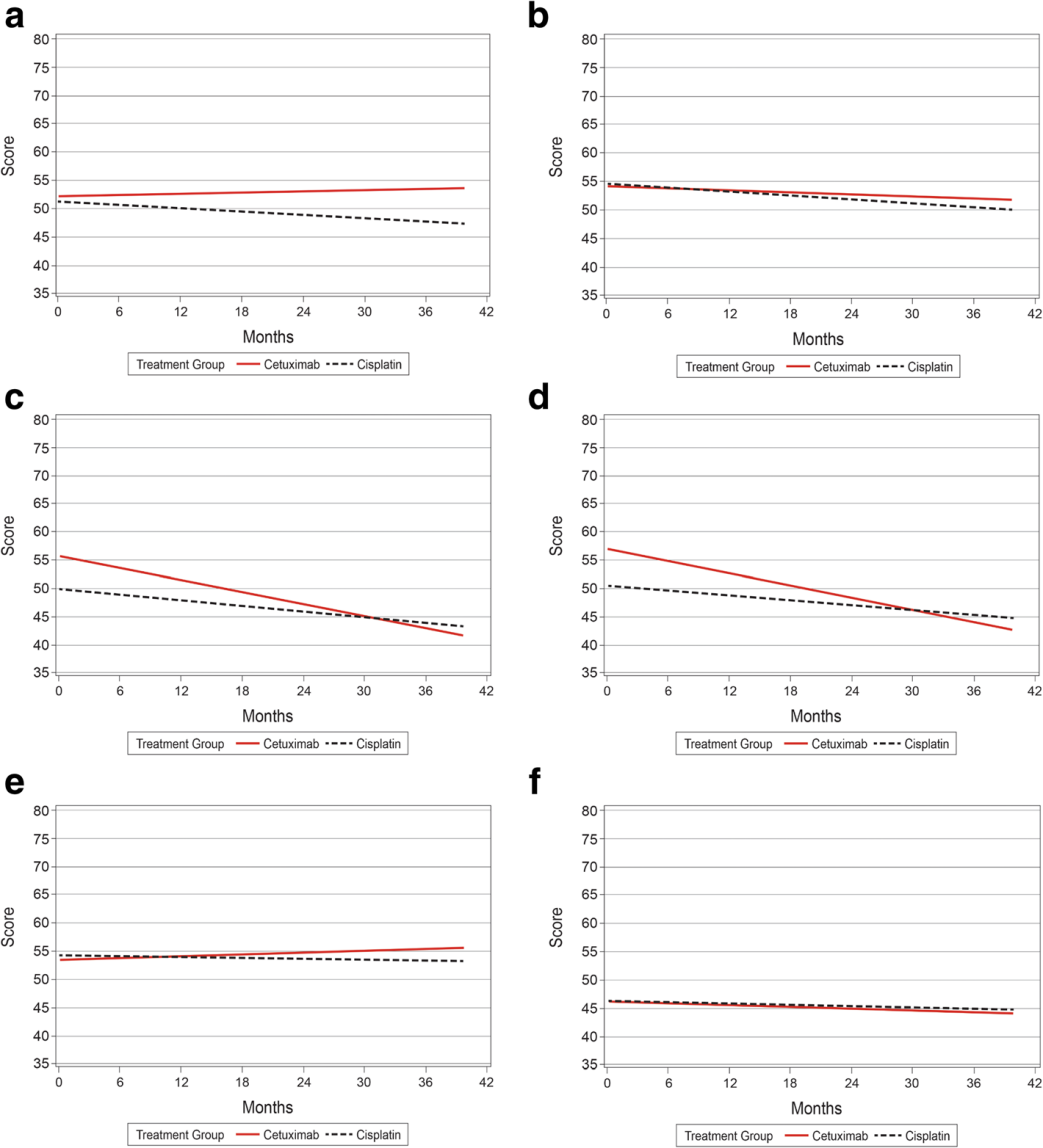
Linear mixed models for **a**) General Physical Symptoms, **b**) Treatment Side Effects, **c**) Acute Distress, **d**) Despair, **e**) Impaired Ambulation, and **f**) Impaired Performance

After controlling for demographic and clinical characteristics using LMM, there was no significant difference between the two cohorts regarding the General Physical Symptoms index score (Fig. 1a). There was, however, a significant effect of time in which the General Physical Symptoms index score decreased (improved) significantly over time (*p* = 0.018) (Table 2). As shown in Fig. 1a, the improvement appears limited to the cisplatin±RT cohort, with scores appearing more stable in the cetuximab±RT cohort. Among other covariates, age at diagnosis (*p* = 0.001) and type of insurance (*p* = 0.010) were significant predictors of the General Physical Symptoms index score. Older age was associated with a slightly lower index score (*p* = 0.001). Compared to patients with private insurance, patients with public insurance (*p* = 0.002) or both public and private insurance (*p* = 0.015), had a statistically and clinically significantly higher (worse) General Physical Symptoms index score.

**Table 2:** Linear mixed models of General Physical Symptoms, Treatment Side Effects, and Acute Distress

	General Physical Symptoms^**a**^		Treatment Side Effects^**b**^		Acute Distress^**c**^	
Effect	Estimate (SE)	*P*-value	Estimate (SE)	*P*-value	Estimate (SE)	*P*-value
Intercept	58.50 (8.141)	< 0.0001	59.60 (5.877)	< 0.0001	60.10 (9.236)	< 0.0001
Time (months) since diagnosis	− 0.10 (0.040)	0.018	− 0.12 (0.024)	< 0.0001	− 0.17 (0.039)	< 0.0001
Treatment: cetuximab±RT (vs. cisplatin±RT)	1.19 (1.667)	0.475	−0.32 (1.221)	0.792	5.77 (2.013)	0.004
Treatment* time since diagnosis	0.13 (0.14)	0.352	0.05 (0.084)	0.540	−0.18 (0.143)	0.205
Age at diagnosis	−0.25 (0.070)	0.001	−0.14 (0.051)	0.005	−0.27 (0.076)	0.001
Male (vs. female)	−2.42 (1.342)	0.072	−2.13 (0.974)	0.029	−6.88 (1.487)	< 0.0001
White (vs. minority/unknown)	0.41 (1.212)	0.733	−0.41 (0.883)	0.644	3.74 (1.347)	0.006
Impaired composite performance status (vs. not impaired)	1.94 (2.438)	0.427	2.01 (1.755)	0.252	−0.13 (2.700)	0.962
Weighted index of comorbid conditions	0.67 (0.641)	0.298	0.57 (0.465)	0.220	1.67 (0.714)	0.020
Smoking status: current or past (vs. never/unknown/undocumented)	2.64 (1.523)	0.083	0.40 (1.102)	0.715	3.98 (1.687)	0.019
Alcohol status	–	0.534	–	0.176	–	0.055
Current or past abuse (vs. never/unknown/undocumented)	0.89 (1.591)	0.576	1.05 (1.153)	0.363	2.09 (1.761)	0.235
Current or past use (vs. never/unknown/undocumented)	−0.66 (1.302)	0.614	−0.82 (0.944)	0.386	−1.61 (1.44)	0.262
Stage at start: Stage III (vs. Stage IV)	0.11 (1.156)	0.922	−0.15 (0.836)	0.854	−0.05 (1.273)	0.971
Oral (vs. non-oral)	−1.64 (3.064)	0.592	0.26 (2.249)	0.908	−3.48 (3.491)	0.319
Pharynx (vs. non-pharynx)	−0.04 (2.987)	0.989	1.88 (2.193)	0.393	−0.18 (3.406)	0.959
Larynx (vs. non-larynx)	−0.73 (3.144)	0.817	0.00 (2.314)	0.999	−2.86 (3.581)	0.425
HPV test results (positive vs. non-positive)	2.60 (3.273)	0.428	3.11 (2.354)	0.187	−5.02 (3.686)	0.174
P16 test results (positive vs. non-positive)	1.469 (1.350)	0.277	0.95 (0.977)	0.332	3.17 (1.479)	0.033
Feeding tube placement (vs. not placed)	1.066 (1.158)	0.356	0.866 (0.841)	0.304	1.26 (1.282)	0.327
BMI	−0.05 (0.093)	0.581	0.005 (0.067)	0.942	−0.06 (0.103)	0.540
Insurance	–	0.010	–	0.052	–	0.047
Only public (vs. only private)	4.85 (1.555)	0.002	2.67 (1.128)	0.018	4.50 (1.728)	0.010
Both public and private (vs. only private)	3.54 (1.458)	0.015	1.70 (1.059)	0.109	2.98 (1.617)	0.066
Other/unknown/undocumented (vs. only private)	4.395 (3.468)	0.205	4.43 (2.574)	0.086	5.09 (3.958)	0.199
US region South (vs. non-South)	4.12 (5.476)	0.452	1.167 (3.942)	0.767	3.68 (6.336)	0.561

Reference level for each comparison is in parentheses

*BMI* body mass index, *HPV* human papilloma virus, *RT* radiation therapy, *SE* standard error, *US* United States

^a^Analysis is based on 237 patients and 1859 observations

^b^Analysis is based on 237 patients and 1866 observations

^c^Analysis is based on 237 patients and 1596 observations

*refers to an interaction

#### Treatment side effects

The LMM for Treatment Side Effects index score showed no difference between the cohorts after controlling for demographic and clinical characteristics (Fig. 1b); however, there was a significant effect of time in which the Treatment Side Effects index score decreased (improved) over time (*p* < 0.0001) (Table 2). Among other covariates, age at diagnosis, gender, and type of insurance were significant predictors of Treatment Side Effects index score. Older age (*p* = 0.005) and male patients (*p* = 0.029) were associated with a lower Treatment Side Effects score. Compared to patients with private insurance, patients with public insurance (*p* = 0.018) had a higher Treatment Side Effects index score.

#### Acute distress

The LMM showed that the cetuximab±RT cohort began with a significantly higher mean Acute Distress index score than the cisplatin±RT cohort (*p* = 0.004); however, score for the cetuximab±RT cohort decreased over time (*p*-value for interaction not significant) (Table 2; Fig. 1c). In addition, there was a main effect of time in which the Acute Distress index score decreased significantly (*p* < 0.0001) for both cohorts over time. Among other covariates, older age (*p* = 0.001) and male gender (*p* < 0.0001) were associated with a lower Acute Distress index score, whereas white race (*p* = 0.006), higher weighted index of comorbid conditions (*p* = 0.020), current or past smoker status (*p* = 0.019), positive p16 test results (*p* = 0.033), and public (*p* = 0.010) or both public and private insurance (*p* = 0.066) were associated with a higher score. Differences associated with race, smoking status, p16 test results, and type of insurance were significant enough to be considered clinically meaningful.

#### Despair

The LMM showed that the cetuximab±RT cohort began with a higher mean Despair index score than the cisplatin±RT cohort (*p* = 0.001); however, scores for the cetuximab±RT cohort decreased over time (*p*-value for interaction not significant) (Table 3; Fig. 1d). In addition, there was a main effect of time in which the Despair index score decreased significantly (*p* = 0.003) for both cohorts over time. Among other covariates, age at diagnosis and type of insurance were significant predictors of Despair. Older age was associated with a slightly lower Despair index score (*p* = 0.001). Compared to patients with private insurance, patients with public insurance (*p* = 0.020) and both public and private insurance (*p* = 0.002) had a higher Despair index score. Differences associated with type of insurance were significant enough to be considered clinically meaningful.

**Table 3:** Linear mixed models of Despair, Impaired Ambulation, and Impaired Performance

	Despair^**a**^		Impaired Ambulation^**b**^		Impaired Performance^**c**^	
Effect	Estimate (SE)	*P*-value	Estimate (SE)	*P*-value	Estimate (SE)	*P*-value
Intercept	61.22 (9.271)	< 0.0001	52.86 (8.981)	< 0.0001	41.96 (9.271)	0.0001
Time (months) since diagnosis	− 0.15 (0.048)	0.003	− 0.03 (0.026)	0.256	− 0.04 (0.037)	0.241
Treatment: cetuximab±RT (vs. cisplatin±RT)	6.62 (2.010)	0.001	−0.82 (1.570)	0.602	−0.13 (1.703)	0.941
Treatment* time since diagnosis	−0.21 (0.141)	0.129	0.08 (0.092)	0.390	−0.01 (0.140)	0.930
Age at diagnosis	−0.27 (0.084)	0.001	−0.08 (0.065)	0.249	−0.05 (0.067)	0.477
Male (vs. female)	−1.32 (1.702)	0.438	− 1.63 (1.204)	0.175	−0.45 (1.227)	0.713
White (vs. minority/unknown)	2.91 (1.513)	0.055	−0.40 (1.114)	0.720	1.62 (1.133)	0.152
Impaired composite performance status (vs. not impaired)	−0.62 (2.846)	0.829	3.34 (2.146)	0.120	2.30 (2.193)	0.294
Weighted index of comorbid conditions	0.35 (0.875)	0.689	1.33 (0.570)	0.020	0.77 (0.583)	0.189
Smoking status: current or past (vs. never/unknown/undocumented)	1.90 (1.910)	0.321	1.67 (1.398)	0.231	2.36 (1.429)	0.100
Alcohol status	–	0.424	–	0.307	–	0.357
Current or past abuse (vs. never/unknown/undocumented)	−0.82 (1.984)	0.679	1.01 (1.460)	0.490	0.66 (1.491)	0.660
Current or past use (vs. never/unknown/undocumented)	−2.09 (1.633)	0.201	−0.91 (1.184)	0.445	−1.08 (1.209)	0.370
Stage at start: Stage III (vs. Stage IV)	0.53 (1.377)	0.698	−0.60 (1.058)	0.571	−0.02 (1.076)	0.989
Oral (vs. non-oral)	−4.92 (3.910)	0.209	−0.84 (2.822)	0.766	−0.48 (2.892)	0.869
Pharynx (vs. non-pharynx)	−5.00 (3.557)	0.161	−0.61 (2.788)	0.826	−0.92 (2.851)	0.746
Larynx (vs. non-larynx)	−4.30 (3.702)	0.246	−3.84 (2.959)	0.195	−3.27 (3.022)	0.279
HPV test results (positive vs. non-positive)	−6.18 (4.057)	0.128	0.715 (3.091)	0.817	0.62 (3.177)	0.845
P16 test results (positive vs. non-positive)	0.69 (1.976)	0.728	1.528 (1.194)	0.201	1.46 (1.218)	0.231
Feeding tube placement (vs. not placed)	0.060 (1.451)	0.967	0.54 (1.076)	0.613	1.20 (1.097)	0.273
BMI	0.055 (0.109)	0.612	0.08 (0.086)	0.376	0.12 (0.087)	0.155
Insurance	–	0.014	–	0.008	–	0.001
Only public (vs. only private)	4.57 (1.956)	0.020	4.65 (1.424)	0.001	5.70 (1.452)	< 0.0001
Both public and private (vs. only private)	5.53 (1.793)	0.002	3.20 (1.338)	0.017	3.77 (1.365)	0.006
Other/unknown/undocumented (vs. only private)	1.72 (4.638)	0.711	3.66 (3.308)	0.268	5.54 (3.403)	0.104
US region South (vs. non-South)	3.20 (5.737)	0.577	1.69 (6.750)	0.802	−1.57 (7.014)	0.823

Reference level for each comparison is in parentheses

*BMI* body mass index, *HPV* human papilloma virus, *RT* radiation therapy, *SE* standard error, *US* United States

^a^Analysis is based on 163 patients and 1048 observations

^b^Analysis is based on 218 patients and 1320 observations

^c^Analysis is based on 218 patients and 1311 observations

*refers to an interaction

#### Impaired ambulation

The LMM showed no significant difference between the two cohorts regarding Impaired Ambulation (Table 3; Fig. 1e). There were no significant main or interaction effects in the model. Among other covariates, the weighted index of comorbid conditions (*p* = 0.020) and insurance type (*p* = 0.008) were significant predictors of Impaired Ambulation index score (Table 3). Higher weighted index of comorbid conditions was associated with a higher Impaired Ambulation index score (*p* = 0.020). Compared to patients with private insurance, patients with public insurance (*p* = 0.001) or both public and private insurance (*p* = 0.017) had a higher Impaired Ambulation index score. The effect of insurance type was large enough to be considered clinically meaningful.

#### Impaired performance

The LMM showed no difference between the two cohorts regarding the Impaired Performance index score (Table 3; Fig. 1f). There were no significant main or interaction effects in the model. Among other covariates, type of insurance was a significant predictor of Impaired Performance. Compared to patients with private insurance, patients with public insurance (*p* < 0.0001) or both public and private insurance (*p* = 0.006) had a higher Impaired Performance index score (Table 3; Fig. 1f). The differences associated with the type of insurance were significant enough to be considered clinically meaningful.

### Individual PCM items

#### Trouble swallowing

The LMM results showed a significant effect of time in which the trouble swallowing score significantly decreased (improved) (*p* = 0.001) over time, indicating an improvement in patients’ QoL (Table 4). Among other covariates, feeding tube placement was a significant predictor of trouble swallowing; patients with a feeding tube had higher (worse) scores than patients without a feeding tube (*p* = 0.008).

**Table 4:** Linear mixed models of trouble swallowing, dry mouth, and numbness/tingling

	Trouble swallowing^**a**^		Dry mouth^**b**^		Numbness/tingling^**c**^	
Effect	Estimate (SE)	*P*-value	Estimate (SE)	*P*-value	Estimate (SE)	*P*-value
Intercept	5.03 (2.519)	0.047	2.81 (2.388)	0.242	3.94 (1.791)	0.029
Time (months) since diagnosis	−0.05 (0.014)	0.001	0.04 (0.011)	0.0003	0.02 (0.010)	0.070
Treatment: cetuximab±RT (vs. cisplatin±RT)	−0.11 (0.546)	0.835	0.17 (0.494)	0.736	−0.15 (0.367)	0.677
Treatment* time since diagnosis	0.01 (0.050)	0.842	−0.02 (0.038)	0.605	0.01 (0.035)	0.820
Age at diagnosis	−0.04 (0.022)	0.070	−0.01 (0.021)	0.556	−0.06 (0.016)	< 0.0001
Male (vs. female)	−0.81 (0.417)	0.053	−0.72 (0.399)	0.071	−0.49 (0.300)	0.100
White (vs. minority/unknown)	0.26 (0.378)	0.500	0.44 (0.362)	0.229	−0.05 (0.272)	0.861
Impaired composite performance status (vs. not impaired)	0.97 (0.748)	0.197	−0.11 (0.716)	0.879	0.49 (0.537)	0.359
Weighted index of comorbid conditions	0.22 (0.199)	0.269	0.11 (0.190)	0.557	0.16 (0.143)	0.264
Smoking status: current or past (vs. never/unknown/undocumented)	− 0.32 (0.472)	0.494	0.19 (0.451)	0.677	0.41 (0.339)	0.227
Alcohol status	–	0.292	–	0.074	–	0.683
Current or past abuse (vs. never/unknown/undocumented)	0.44 (0.494)	0.377	−0.32 (0.472)	0.504	0.31 (0.355)	0.387
Current or past use (vs. never/unknown/undocumented)	−0.25 (0.404)	0.540	−0.85 (0.387)	0.028	0.17 (0.291)	0.567
Stage at start: Stage III (vs. Stage IV)	0.30 (0.358)	0.399	−0.15 (0.343)	0.666	0.05 (0.258)	0.862
Oral (vs. non-oral)	0.96 (0.966)	0.319	0.13 (0.917)	0.889	−0.63 (0.685)	0.357
Pharynx (vs. non-pharynx)	1.79 (0.942)	0.058	0.70 (0.894)	0.434	−0.82 (0.667)	0.221
Larynx (vs. non-larynx)	1.06 (0.994)	0.285	−0.07 (0.944)	0.941	−0.79 (0.704)	0.261
HPV test results (positive vs. non-positive)	0.61 (1.005)	0.541	1.52 (0.957)	0.112	0.46 (0.719)	0.526
P16 test results (positive vs. non-positive)	−0.24 (0.418)	0.564	0.38 (0.401)	0.344	0.60 (0.303)	0.048
Feeding tube placement (vs. not placed)	0.95 (0.360)	0.008	0.77 (0.345)	0.026	−0.12 (0.259)	0.651
BMI	−0.03 (0.029)	0.380	0.05 (0.028)	0.107	0.01 (0.021)	0.618
Insurance	–	0.180	–	0.083	–	0.026
Only public (vs. only private)	0.64 (0.484)	0.184	0.94 (0.462)	0.043	0.89 (0.347)	0.011
Both public and private (vs. only private)	0.10 (0.454)	0.836	−0.004 (0.434)	0.993	0.87 (0.326)	0.008
Other/unknown/undocumented (vs. only private)	2.04 (1.113)	0.067	1.50 (1.054)	0.171	0.36 (0.785)	0.644
US Region South (vs. non-South)	0.22 (1.693)	0.897	−0.44 (1.591)	0.783	0.79 (1.190)	0.508

Reference level for each comparison is in parentheses

*BMI* body mass index, *HPV* human papilloma virus, *RT* radiation therapy, *SE* standard error, *US* United States

^a^Analysis is based on 237 patients and 1848 observations

^b^Analysis is based on 237 patients and 1857 observations

^c^Analysis is based on 237 patients and 1851 observations

*refers to an interaction

#### Dry mouth

The LMM results showed a significant main effect of time, with the dry mouth score decreasing (improving) over time (*p* = 0.0003) (Table 4). Among other covariates, public insurance (*p* = 0.043) and feeding tube placement (*p* = 0.026) were associated with a slight increase in score, whereas current or past alcohol use (*p* = 0.028) was associated with a slight decrease.

#### Numbness and tingling

The LMM results showed no effect with the passage of time on numbness and tingling, but showed that age at diagnosis, p16 test results, and type of insurance were significant predictors of numbness/tingling (Table 4). Older age was associated with a significantly lower (better) numbness/tingling score (*p* < 0.0001). Patients with positive p16 test results had a slightly higher numbness/tingling score than patients with negative or undocumented p16 test results (*p* = 0.048). Compared to patients with private insurance, patients with public insurance (*p* = 0.011) or both public and private insurance coverage (*p* = 0.008) had a higher numbness/tingling score.

#### Rash

The LMM showed a significant main effect of treatment cohort, and a significant interaction of treatment cohort and time since diagnosis (Table 5). Patients in the cetuximab±RT cohort had higher rash scores (*p* < 0.0001) than the cisplatin±RT cohort at baseline, but the interaction shows that rash scores in the cetuximab±RT cohort decreased significantly over time compared with the cisplatin±RT cohort (*p* = 0.013) (Table 5).

**Table 5:** Linear mixed models of rash, weight loss, and mouth sores/ulcers

	Rash^**a**^		Weight loss^**b**^		Mouth sores/ulcers^**c**^	
Effect	Estimate (SE)	*P*-value	Estimate (SE)	*P*-value	Estimate (SE)	*P*-value
Intercept	1.38 (1.065)	0.198	5.051 (2.337)	0.032	5.661 (1.795)	0.002
Time (months) since diagnosis	−0.01 (0.007)	0.299	− 0.05 (0.016)	0.002	− 0.06 (0.011)	< 0.0001
Treatment: cetuximab±RT (vs. cisplatin±RT)	1.45 (0.235)	< 0.0001	0.12 (0.511)	0.817	−0.24 (0.412)	0.567
Treatment* time since diagnosis	−0.06 (0.023)	0.013	−0.04 (0.047)	0.356	0.03 (0.036)	0.375
Age at diagnosis	−0.03 (0.009)	0.005	−0.05 (0.021)	0.036	−0.05 (0.015)	0.001
Male (vs. female)	−0.11 (0.179)	0.541	− 0.31 (0.434)	0.480	−0.59 (0.291)	0.044
White (vs. minority/unknown)	−0.01 (0.164)	0.946	−0.41 (0.387)	0.291	0.35 (0.268)	0.188
Impaired composite performance status (vs. not impaired)	0.29 (0.314)	0.363	0.88 (0.713)	0.216	−0.53 (0.506)	0.291
Weighted index of comorbid conditions	−0.01 (0.085)	0.896	0.02 (0.222)	0.927	−0.06 (0.141)	0.697
Smoking status: current or past (vs. never/unknown/undocumented)	−0.20 (0.202)	0.334	0.24 (0.488)	0.628	0.04 (0.332)	0.912
Alcohol status	–	0.061	–	0.361	–	0.074
Current or past abuse (vs. never/unknown/undocumented)	0.49 (0.212)	0.020	0.61 (0.506)	0.227	0.72 (0.346)	0.037
Current or past use (vs. never/unknown/undocumented)	0.28 (0.174)	0.105	−0.02 (0.420)	0.970	0.09 (0.283)	0.750
Stage at start: Stage III (vs. Stage IV)	−0.04 (0.154)	0.773	−0.24 (0.353)	0.493	0.10 (0.250)	0.699
Oral (vs. non-oral)	−0.04 (0.414)	0.922	−0.55 (1.017)	0.588	0.82 (0.699)	0.243
Pharynx (vs. non-pharynx)	−0.19 (0.404)	0.637	−0.32 (0.923)	0.733	0.66 (0.682)	0.336
Larynx (vs. non-larynx)	−0.20 (0.428)	0.639	−0.09 (0.963)	0.929	−0.41 (0.719)	0.567
HPV test results (positive vs. non-positive)	0.14 (0.426)	0.740	0.54 (1.007)	0.591	1.52 (0.712)	0.033
P16 test results (positive vs. non-positive)	0.05 (0.181)	0.776	0.06 (0.512)	0.911	−0.24 (0.293)	0.414
Feeding tube placement (vs. not placed)	−0.05 (0.156)	0.739	−0.14 (0.373)	0.707	0.42 (0.254)	0.099
BMI	0.02 (0.012)	0.222	−0.02 (0.028)	0.583	−0.01 (0.020)	0.777
Insurance	–	0.042	–	0.475	–	0.375
Only public (vs. only private)	0.07 (0.209)	0.738	0.61 (0.500)	0.226	0.39 (0.343)	0.261
Both public and private (vs. only private)	0.53 (0.196)	0.008	0.65 (0.459)	0.156	0.42 (0.320)	0.185
Other/unknown/undocumented (vs. only private)	0.19 (0.496)	0.700	0.63 (1.235)	0.612	1.03 (0.833)	0.215
US Region South (vs. non-South)	0.25 (0.706)	0.727	0.86 (1.428)	0.545	−1.14 (1.225)	0.354

Reference level for each comparison is in parentheses

BMI, body mass index; HPV, human papilloma virus; RT, radiation therapy; SE, standard error; US, United States

^a^Analysis is based on 237 patients and 1856 observations

^b^Analysis is based on 163 patients and 1303 observations

^c^Analysis is based on 237 patients and 1852 observations

*refers to an interaction

Among other covariates, age at diagnosis, alcohol abuse, and insurance type were significant predictors of rash. Older age was associated with a slightly lower score (*p* = 0.005); while patients with a record of (current or past) alcohol abuse (*p* = 0.020) and patients with both public and private insurance coverage (*p* = 0.008) had a higher rash score.

#### Weight loss

The LMM showed a significant effect of time in which the weight loss score decreased over time (*p* = 0.002) (Table 5). Among other covariates, older age was associated with a significantly lower score (*p* = 0.036).

#### Mouth sores and ulcers

The LMM results showed a significant effect of time in which the score for mouth sores and ulcers decreased over time (*p* < 0.0001) (Table 5). Among other covariates, age at diagnosis, gender, alcohol abuse, and HPV results were significant predictors of mouth sores and ulcers. Older age (*p* = 0.001) and male gender (*p* = 0.044) were associated with a slightly lower score. In contrast, current or past alcohol abuse (*p* = 0.037) and positive HPV result (*p* = 0.033) were associated with a higher score. The effect of HPV-positivity was large enough to be considered clinically meaningful.

## Discussion

This study was a retrospective, observational study that examined the characteristics and QoL experience of patients receiving cetuximab±RT vs. cisplatin±RT for treatment of locoregionally advanced SCCHN. After application of propensity score weighting, the weighted cohorts appeared demographically and clinically similar. The QoL experience of patients across cohorts was also comparable, except that cetuximab±RT patients appeared to begin therapy with more severe symptoms of Acute Distress and Despair. Cetuximab±RT patients appeared to experience more severe rash symptoms, consistent with the known toxicity profile for cetuximab.

This study provides new information regarding QoL outcomes among patients with advanced SCCHN treated with cetuximab or cisplatin in the community oncology setting. Notably, it documents generally improving symptomatology regarding treatment side effects, and regarding psychological symptoms of distress and despair over time. The study also showed that cetuximab patients had significantly higher baseline Acute Distress and Despair compared to the cisplatin patients; although Acute Distress and Despair index scores improved more quickly over time for cetuximab patients.

The finding of higher baseline Acute Distress and higher baseline Despair PCM index scores among cetuximab patients was unexpected. However, if cetuximab was viewed as more tolerable than cisplatin, oncologists may have offered it to patients who were more overtly distressed, or struggling generally in ways that reflected underlying distress—a selection effect in who was offered cetuximab. This finding aligns with previous observations that oncologists may have offered oral chemotherapies rather than more demanding infused therapies to depressed patients [22].

In this study, patients with private insurance tended to fare better on several QoL endpoints compared to patients with public insurance or both public and private insurance. These QoL endpoints included general physical symptoms, treatment side effects, acute distress, despair, impaired ambulation, and impaired performance. The insurance effect observed in this study may partly be attributed to the younger age of privately insured patients (< 65 years) compared to the age of patients covered under public insurance (≥65 years) in the US.

Other findings regarding selected patient-reported symptoms align with expectations given the toxicity profiles of the respective therapies. This includes the finding of higher scores for numbness and tingling among cisplatin±RT patients, and higher rash scores in the cetuximab±RT cohort, although rash appeared more transient for the cetuximab patients, with significant improvement over time.

Findings from this study may suggest more about how patients are selected for therapy than about differences in the QoL impacts of the respective treatments. The broad pattern of findings suggests that patients treated for SCCHN in the locoregionally advanced setting may experience moderate improvement in several QoL dimensions over time, including those related to treatment side effects and psychological functioning, especially symptoms of anxiety and depression. Differences in outcomes among patients receiving cetuximab±RT vs. cisplatin±RT did not differ greatly except in selected symptoms known to be associated with the treatments.

The findings also suggest that patients with more severe psychological distress at the outset may have been offered what was viewed as a less toxic treatment, and that they experienced greater improvement in QoL over the course of that treatment. Although the findings do not point to a causal relationship, clinicians may wish to consider the expected toxicities of treatment alternatives in light of the psychological and QoL status of patients as they embark on treatment in this setting.

This study has several limitations. First, the PCM survey was not designed to be specific to the experience of head and neck cancer patients, and may have missed key dimensions of QoL relevant to this population. The use of a more targeted instrument such as the European Organisation for Research and Treatment of Cancer Quality of Life Head and Neck module (EORTC QLQ-H&N35) might have allowed for the capture of more nuanced symptoms experienced by head and neck cancer patients. Second, the study did not categorize cisplatin-based therapy into high-dose vs. low-dose cisplatin for comparative analysis. As per the National Comprehensive Cancer Network treatment guidelines, CRT with high-dose every-3-week cisplatin is preferred over low-dose weekly cisplatin; however, older, sicker, and poor-performing patients who are unable to tolerate the high-dose cisplatin are often treated with low-dose weekly cisplatin or cetuximab [11]. Breakdown of cisplatin patients into high and low dose cohorts might have yielded additional interesting findings. A third limitation is that the sample size of the cetuximab±RT cohort with PCM data was much smaller than that of the cisplatin±RT cohort, even after rescaling of the weights. The implication of the difference in sample sizes of the two cohorts is likely to be one of statistical power, but it may have also affected the pattern of findings in unknown ways. Finally, the analysis was limited to patients initiating treatment for locoregionally advanced disease in the community oncology setting. Findings from this study may not generalize to patients treated exclusively in the metastatic setting, or to those who receive their care in non-community-based settings.

## Conclusion

SCCHN is among the most debilitating cancers due to the effect of the tumor and its treatment on the physiological functions of swallowing, speech, and breathing, and the impact of treatment-related facial disfigurement [29]. Consideration of QoL in this patient population is, therefore, especially important. Findings from this study showed some differences in the QoL impacts of patients receiving cetuximab±RT and cisplatin±RT treatments, but also highlighted patterns of improvement in QoL over time across treatments. These findings will aid healthcare professionals in making informed treatment decisions that take into consideration QoL outcomes associated with different regimens in the care of locoregionally advanced SCCHN.

## Data Availability

Contractual provisions around use of the data from source practices prevent us from making the data publicly available. Individual requests will be handled on a case by case basis.

## Abbreviations

BMI: Body mass index
CRT: Chemoradiation therapy
ECOG: Eastern Cooperative Oncology Group
HPV: Human papillomavirus
IPTW: Inverse probability of treatment weights
LMM: Linear mixed models
PCM: Patient Care Monitor
PSM: Propensity score model
QoL: Quality of life
SCCHN: Squamous cell carcinoma of the head and neck
±RT: With or without radiation therapy
US: United States
